# Caspase-Mediated Truncation of Tau Potentiates Aggregation

**DOI:** 10.1155/2012/731063

**Published:** 2012-09-04

**Authors:** Sangmook Lee, Thomas B. Shea

**Affiliations:** Center for Cellular Neurobiology and Neurodegeneration Research, Department of Biological Sciences, University of Massachusetts, One University Avenue, Lowell, MA 01854, USA

## Abstract

Caspase-mediated truncation of tau is associated with aggregation. We examined the impact of manipulation of caspase activity on intracellular aggregation of a mutant form of tau (3PO) that forms spontaneous aggregates. Treatment with the caspase inhibitor Z-VAD-fmk reduced both N and C-terminal tau truncation but did not significantly reduce aggregation. Treatment with staurosporine, which activated caspases, increased C-terminal but not N-terminal truncation and enhanced aggregation. These findings suggest that caspase activation is one potential route, rather than an obligatory initiation step, in aggregation, and that N- and C-terminal truncation contribute differentially to aggregation.

One pathological hallmark of tauopathies is aggregation of the microtubule-associated protein tau. A growing body of evidence highlights the importance of truncation in initiation and potentiation of tau aggregation [1–8]. Tau truncated at amino acid D421 has been detected in Alzheimer's disease (AD) [3, 4, 9, 10] and other tauopathies [11]. C-terminal truncation of tau introduces a conformational change, along with phosphorylation, contributes to aggregation [12–14].

Caspases are serine-aspartyl proteases typically considered to be activated during apoptosis, but can also be activated without apoptosis [15]. In this regard, while caspase activation precedes and promotes tangle formation [1], tangle-bearing neurons can survive for extended periods [16, 17]. Cleavage of tau at D421 has been suggested to be mediated by caspase-3 [3, 4]. By contrast, analyses of transgenic mice suggest that caspase-6, rather than caspase-3, may truncate tau at D421 [8]. Additional analyses in mice suggest that caspase activation may not be obligatory for tangle initiation, but rather may represent one of multiple mechanisms contributing to tau aggregation [18]. Truncation of tau at D13, which can also be mediated by caspase-6 [19], has been detected in AD brains [20]. Whether caspase-6 mediates this cleavage *in situ* is unclear [20, 21]. Any role for N-terminal cleavage in aggregation remains to be elucidated.

Herein, we present evidence that tau truncation at D421 is not necessarily mediated by caspase-3 and that neither N- nor C- terminal truncation is essential for aggregation.

NB2a/d1 cells were cultured and transfected as described [22] with a plasmid expressing GFP (Green Fluorescent Protein) tagged 3 PO tau, a mutated form of human tau that spontaneously aggregates but retains its microtubule binding capacity (gift of Dr. F. S. Wouters, Max-Planck-Institute, Germany) [23]. Medium containing plasmid was left on the cultures for 12 hrs, after which the medium was changed and cultures were incubated an additional 60 hrs to allow for accumulation of GFP-3PO [22]. To activate caspases, cells received 1 *μ*M staurosporine (Cell Signaling, Beverly, MA) for the final 4 hrs of incubation [24]. To inhibit caspase activity, the pan-caspase inhibitor, Z-VAD-fmk (Bnzyloxycarbonyl-Val-Ala-Asp(OMe)-fluoromethylketone; 100 *μ*M; Tocris Bioscience, Ellisvile, MS), was added 12 hrs after transfection and maintained until cell harvest at 72 hrs [25, 26].

Cells were fixed and reacted overnight with rabbit anti-GFP (Invitrogen, Grand Island, NY) and mouse monoclonal antibodies Tau12 (directed at aa9–18; EMD Millipore, Temecula, CA), Tau46 (directed at aa404–441; Upstate BioChemical, Lake Placid, NY) [27] or C3 (which detects tau cleaved at D421; Santa Cruz Biotechnology, Santa Cruz, CA) [3] as described [22].

Cultures were washed with PBS, incubated with Alexa-488 conjugated and Rhodamine Red-X conjugated secondary antibodies for 1 hr at room temperature. Quantification was performed using Image J (http://rsbweb.nih.gov/ij/) on captured images [22]. Approximately 100 transfected cells (confirmed by presence of GFP) were quantified per condition for 3 independent experiments. The immunofluorescent intensity of aggregates and adjacent regions of the cytoplasm of equivalent size were compared for approximately 40 cells per condition for 3 independent experiments. Values were presented as the mean percentile ± the standard error of the mean (SEM).

Cells were washed twice with ice-cold PBS and scraped from the plate in 10 mM Tris-HCl (pH 7.4) containing 1% Triton-X 100, 100 mM NaCl, 1 mM EGTA, 1 mM EDTA, 10% Glycerol, 0.1% SDS, 0.5% deoxycholate, deoxyribonuclease I, and protease and phosphatase inhibitor cocktails (Roche Applied Science, Indianapolis, IN) at 4°C. Lysates were obtained by sonication followed by centrifugation (13,000×*g* for 20 min). Samples were normalized according to total protein and subjected to SDS-polyacrylamide gel electrophoresis and transferred onto PVDF membranes. Membranes were blocked with 5% BSA and 5 mM sodium fluoride in TBST (0.1% Tween 20 in TBS) for 1 hr then incubated overnight at 4°C with anti-GFP, Tau46, C3, Tau12, Tau5 (which reacts with aa 210–241; gift of Dr. Lester Binder, Northwestern University, IL), an antibody directed against cleaved caspase-3 (since activation requires cleavage of its 35 kDa zymogen into activated 17 kDa and 19 kDa fragments; Cell Signaling, Beverly, MA) [26], and an antibody directed against the caspase-3 substrate, Poly ADP-ribose polymerase (PARP, Cell Signaling, Beverly, MA) [26, 28]. Membranes were washed with the same buffer, incubated with alkaline phosphatase-conjugated secondary antibodies for 1 hr at room temperature, then developed using a NBT/BCIP substrate kit (Promega Madison, WI). Densitometric levels of immunoreactive species were quantified for 3 separate immunoblots, each derived from an independent experiment, using Image J.

Trace levels of cleaved caspase-3 was present in untreated cells, consistent with basal levels of caspase activity. Staurosporine increased the level of cleaved caspase-3 by 34%, and increased cleavage of the known caspase-3 substrate, PARP, by 3-fold compared to those of untreated cells. The pan-caspase inhibitor, Z-VAD-fmk, did not decrease steady-state cleavage products of caspase-3 and PARP, but prevented the cleavage of the intact PARP by 21%, suggesting the presence of activated caspases prior to the addition of the inhibitor. Co-incubation with staurosporine and Z-VAD-fmk attenuated staurosporine-induced caspase activity, confirming inhibition of caspase-3 activity by Z-VAD-fmk (Figures 1(a) and 1(b)).

Overall Tau levels were also slightly reduced by staurosporine, with the most extensive loss of the slowest-migrating isoforms.Treatment with Z-VAD-fmk did not significantly increase Tau levels but prevented the depletion of slowest-migrating isoforms, indicating that their depletion was the result of caspase-mediated truncation of one or both termini (Figures 1(a) and 1(b)).

Consistent with prior studies [23], apoptosis was not detected within our total 72 hrs of incubation posttransfection in untreated cells, nor was it induced by treatment with either and pharmacological agent (Figure 1(c)).

We next considered how these pharmacological treatments affected tau cleavage. Tau has multiple cleavage sites within both termini [29] (Figure 2(a)). We therefore compared levels of GFP (for the N-terminus) and T46 or C3 (for the C-terminus) with the centrally-situated Tau5 epitope to provide an index of truncation at either terminal (Figure 2(b)).

N-terminal truncation was monitored by generating a ratio of GFP/Tau5. This ratio increased following treatment with Z-DAV-fmk, indicating that tau undergoes steady-state caspase-mediated N-terminal cleavage in untreated cells. No increase in N-terminal truncation was noted following staurosporine treatment (Figure 2(b)), suggesting that the caspase(s) activated by short-term (4 hrs) staurosporine treatment do not mediate N-terminal cleavage under these conditions. The presence of multiple low molecular weight GFP-reactive fragments (i.e., retaining their full N-terminal region) regardless of manipulation of caspase activity (Figure 2(b)) suggests that proteases other than caspases mediate progressive C-terminal truncation.

An index of C-terminal truncation was provided by the ratio of Tau46/Tau5 and C3/Tau5. Staurosporine dramatically increased C3 immunoreactivity, confirming tau cleavage at D421 [3]. Unexpectedly, Z-VAD-fmk increased C3 immunoreactivity. One potential explanation is that, since Z-VAD-fmk is a pan-caspase inhibitor, it may have differentially inhibited multiple proteolytic events, such as truncation of tau by caspase-6 at D402 (Figure 2(a)), which would preserve C3 immunoreactivity generated by cleavage at D421. A second, nonexclusive possibility is that cleavage at D421 is not solely mediated by caspase-3. Staurosporine decreased Tau46 immunoreactivity while Z-VAD-fmk increased it; these alterations were anticipated, since truncation at D421 eliminates the more distal Tau46 epitope (see Figure 2(a)).

To determine whether or not manipulation of tau truncation influenced aggregation, we quantified the percentage of cells displaying aggregates and the number of aggregates per cell. Staurosporine increased the percentage of cells containing aggregates and the number of aggregates per cell (Figures 3(a), and 3(b)). By contrast, Z-VAD-fmk did not significantly reduce the percentage of cells with aggregates, nor did it significantly reduce the number of aggregates per cell. These findings indicate that caspase activation enhances aggregation but may not be essential for aggregation.

Finally, we compared the relative levels of truncated tau within aggregates versus the surrounding cytoplasm. Since we added Z-VAD-fmk shortly after transfection and maintained it until harvest, aggregates in Z-VAD-fmk-treated cells would have formed under conditions of caspase inhibition. Since staurosporine was added for the final 4 hrs prior to harvest, aggregation had commenced prior to staurosporine treatment, allowing us to evaluate the effect of caspase activation on existing aggregates. The distribution of GFP and Tau46 within aggregates and the adjacent aggregate-free cytoplasm did not differ significantly among untreated cells and those treated with either Z-VAD-fmk or staurosporine, confirming that similar levels of tau containing intact N and C termini were incorporated into aggregates independent of caspase manipulation (Figure 3(c)). Consistent with immunoblot analyses (Figure 2(b)), marginal C3 immunoreactivity was detected in untreated and Z-VAD-fmk-treated cells and was evenly distributed between aggregates and the surrounding cytoplasm (Figure 3(c)). Staurosporine dramatically increased C3 immunoreactivity, the majority of which localized within aggregates (Figure 3(c)). Notably C3 immunoreactivity was prominent along the periphery of aggregates, while GFP and T46 were evenly distributed throughout the entire aggregate (Figure 3(c)). These immunofluorescent observations collectively suggest that, tau truncated at D421 is incorporated into existing aggregates and that aggregates can form in its absence.

Herein, we utilized GFP as an N-terminal marker of tau. Since the GFP tag itself could be cleaved and may therefore compromise interpretation, we utilized the additional N-terminal specific antibody Tau12 (which recognizes an epitope at aa9–18). Comparative immunoblot analyses with anti-GFP and Tau12 did not reveal anti-GFP specific band(s) which were absent in Tau12 blots, regardless of manipulation of caspase activity (Figure 4(a)). Staurosporine treatment decreased tau levels compared to untreated cells as indicated by both GFP and Tau12. Treatment with Z-VAD-fmk statistically increased tau levels versus untreated cells as indicated by anti-GFP and displayed a trend towards significance as indicated by Tau12 (*P* = 0.06, Student's* t*-test). Immunofluorescent analyses with anti-GFP and Tau 12 revealed identical tau distribution within aggregates and cytosol under all conditions (Figure 4(b)). These findings support the validity of anti-GFP as an N-terminal marker of tau under the conditions utilized herein.

Our findings indicate that one or more caspases are involved in truncation of both tau termini but, consistent with other studies [8], indicate that other proteases may be involved in progressive truncation. We also provide evidence that truncation may not be essential for aggregation but rather that caspase-mediated cleavage at D421 enhances aggregation and promotes incorporation of tau into aggregates. These findings suggest that caspase activation is one potential route, rather than an obligatory initiation step, in neurofibrillary tangle formation and enlargement [18, 30].

## Figures and Tables

**Figure 1:**
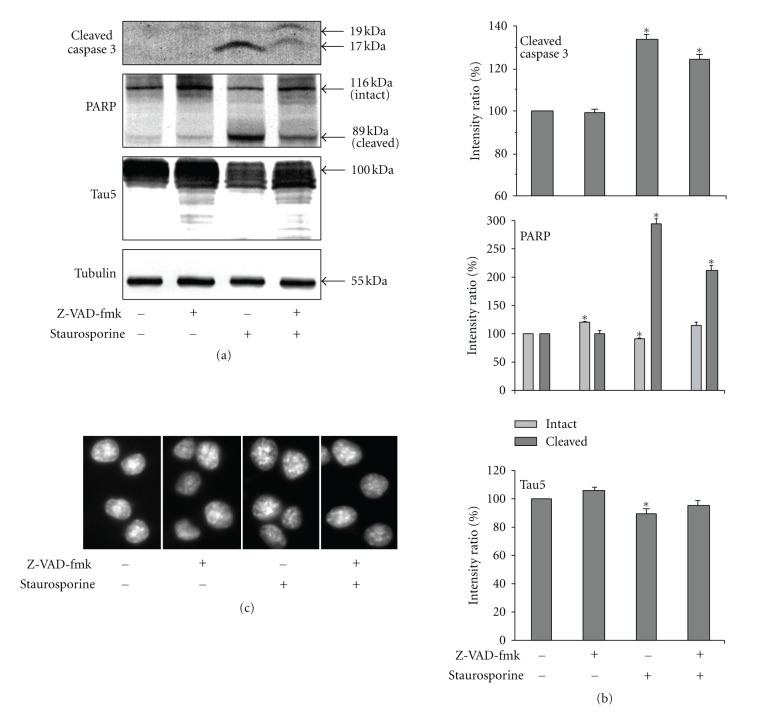
Pharmacological manipulation of caspase activity. (a) presents immunoblots of lysates from cells treated with Z-VAD-fmk, staurosporine, or both, along with untreated controls, probed with antibodies directed against cleaved caspase-3, PARP, tau, and tubulin (as a loading control) as indicated. The accompanying graphs (b) present the mean intensity ratio (±SEM) normalized to untreated controls from 3 immunoblots. Note increased levels activated caspase-3 and PARP cleavage, and reduced levels of tau following staurosporine treatment. Note that Z-VAD-FMK did not reduce steady-state levels of cleaved caspase-3, cleaved PARP, or tau but increased intact PARP (21%), suggesting the presence of ongoing caspase activity and PARP cleavage in these cells prior to treatment. Co-treatment with Z-VDA-fmk attenuated staurosporine-induced caspase activity, PARP, and tau cleavage. The percentage changes were quantified by densitometric analyses of immunoblots. An asterisk indicates statistical significance when compared to untreated controls (*P* < 0.05, Student's* t*-test). (c) presents representative images of DAPI-stained cells. Note presence of healthy, nonapoptotic nuclei, indicating that incubation with staurosporine for 4 hours did not induce apoptosis.

**Figure 2:**
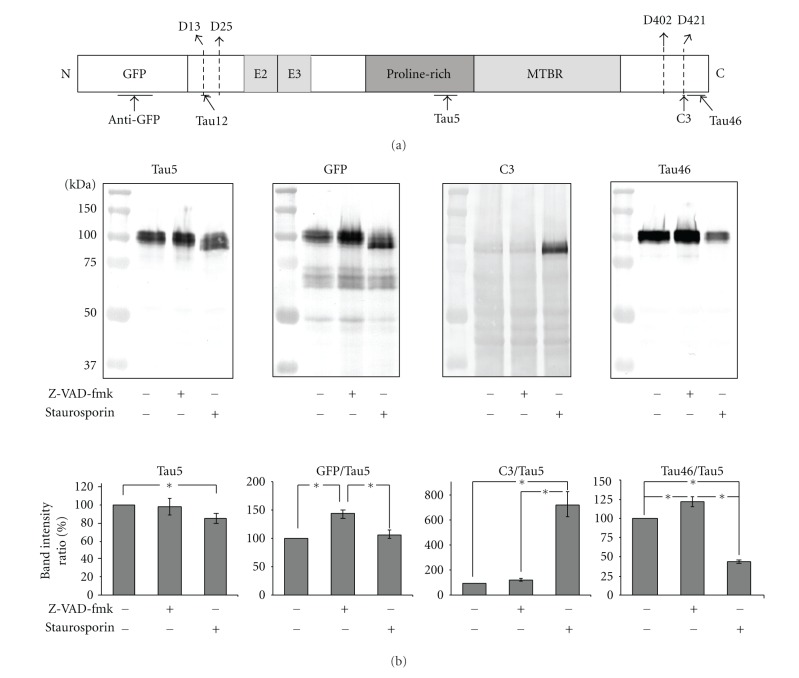
Tau truncation by caspases. (a) depicts GFP-tagged 3 PO tau. The caspase cleavage sites reported in AD brains and the epitopes recognized by the antibodies used for the truncation detection in this study are noted. GFP tagged 3PO tau is depicted. Caspase-3 has been reported to cleave tau at D25 and D241. Caspase-6 has been reported to cleave tau at D13, D402, or D421. (b) presents representative immunoblots of lysates from cell expressing GFP-3PO with and without treatment with staurosporine or Z-VAD-fmk as indicated. Migration of molecular weight standards is indicated on the left of each blot. The accompanying graphs presents the mean intensities (±SEM) derived from 4 independent immunoblots. The relative intensities of the uppermost bands from anti-GFP, C3, and Tau46 were normalized to those of Tau5; lower-molecular weight breakdown products were not considered in these densitometric analyses. An asterisk indicates statistical significance (*P* < 0.05, Student's* t* test).

**Figure 3:**
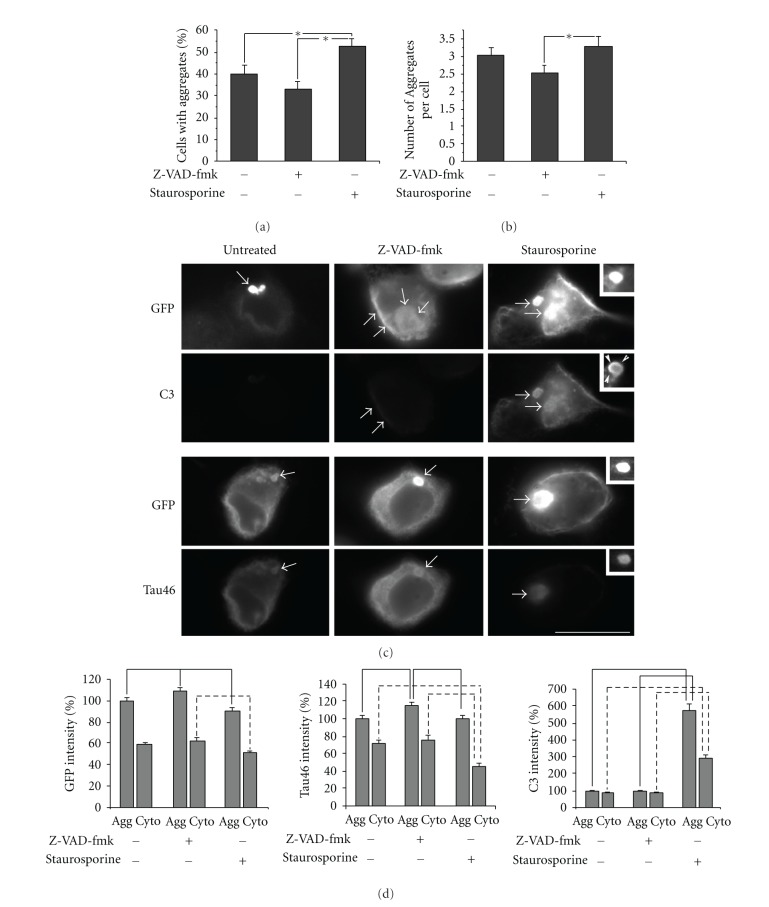
Effect of Z-VAD-fmk and staurosporine on aggregation and truncation. (a) presents quantification of the percentage of cells containing aggregates as detected by GFP with and without pharmacological manipulation of caspase activity. (b) presents quantification of the number of aggregates per cell. Note that staurosporine significantly increased, while Z-VAD-fmk induced a detectable but nonstatistically significant decrease, the percentage of cells with aggregates as well as the number of aggregates per cell. Values represent the mean ± SEM from 3 independent experiments (*P < 0.05, Student's* t*-test). (c) presents double immunofluorescent staining with anti-GFP and either C3 (upper images) or Tau46 (lower images) with and without treatment with Z-VAD-fmk or staurosporine as indicated. The bar represents 20 *μ*m. Arrows indicate representative aggregates. Note that staurosporine-treated cells display stronger C3 immunoreactivity within aggregates and very weak Tau46 immunoreactivity within the cytosol. Staurosporine panels include insets from a different transfected cell to highlight that C3, but not Tau46 or GFP, is localized around the periphery of some aggregates. (d) presents quantification of immunofluorescence intensity of anti-GFP, C3 and Tau46 between aggregates and adjacent cytosolic tau in the same cells. All bars connected by lines are statistically different (*P* < 0.05; Student's* t*-test); continuous lines connect values for aggregates, while dotted lines connect values for cytosol. Values represent the mean ± SEM (*P* < 0.05, Student's* t*-test).

**Figure 4:**
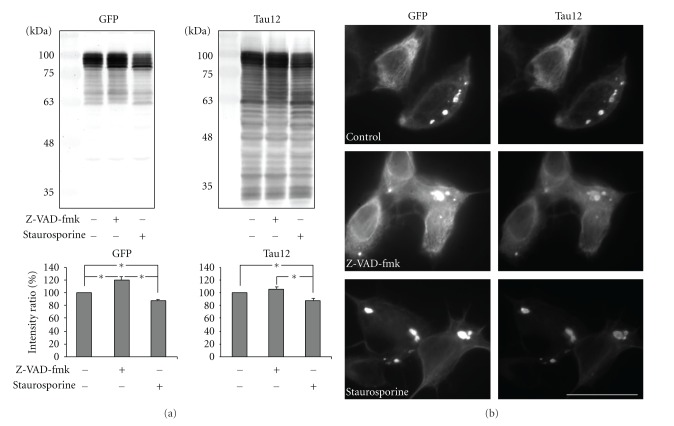
Validity of GFP as an N-terminal marker of tau. (a) presents representative immunoblots of lysates from cell expressing GFP-3PO without any treatment and with treatment of either staurosporine or Z-VAD-fmk as indicated. Blots were probed with anti-GFP or Tau12 (aa9–18) to show N-terminal truncation of tau upon pharmacological manipulation of caspase activity. Migration of molecular weight standards is indicated on the left of each blot. Note the absence of anti-GFP specific band(s) absent in Tau12 blots. The accompanying graphs presents the mean intensities (±SEM) derived from 3 immunoblots. The relative intensities of the uppermost bands from anti-GFP and Tau12 were normalized to that of their respective untreated controls. Staurosporine decreased tau levels in both GFP and T12 blots. Z-VAD-fmk statistically increased tau levels as indicated by anti-GFP and displayed a trend towards significance by Tau 12 (*P* = 0.062, Student* t*-test). An asterisk indicates a statistical difference (*P* < 0.05; Student's *t*-test). (b) presents double immunofluorescent staining of cells untreated or treated with either Z-VAD-fmk or staurosporine as indicated. The bar represents 20 *μ*m. Note the identical colocalization pattern between anti-GFP and Tau12 stainings regardless of pharmacological manipulation of caspase activity.
